# The implications of hyperekplexia on children’s quality of life: a report on two cases

**DOI:** 10.1590/1984-0462/2025/43/2024189

**Published:** 2025-03-24

**Authors:** Beatriz Salimon Carlos dos Santos, João Pedro Garcia de Mattos, Laura Souza Juliano, Rodrigo Rigoleto de Souza, César Antônio Franco Marinho

**Affiliations:** a Centro Universitário de Adamantina, Adamantina, SP, Brazil.

**Keywords:** Hyperekplexia, Startle reflex, Neurodevelopmental disorders, Hiperecplexia, Reflexo de sobressalto, Transtornos do neurodesenvolvimento

## Abstract

**Objective:**

To report two pediatric cases of hyperekplexia in a small city of São Paulo state, Brazil.

**Case description:**

Two female patients, one aged three years and six months and one aged five months, receiving care from an APAE (Association of Parents and Friends of People with Disabilities) unit, were diagnosed with hyperekplexia 1, a neurological disorder characterized by an excessive startle response. Hyperekplexia cases can be divided into three subgroups: hereditary, sporadic, and symptomatic. Several specialists have examined patient 1 since she was three weeks old, leading to two initial diagnostic hypotheses (childhood chronic non-progressive encephalopathy and spastic cerebral palsy). She was diagnosed with hyperekplexia 1 at eleven months when a genetic test revealed changes in the GLRA1 gene. Patient 2, at birth, presented hyperextension of both legs, low-set ears, cranial asymmetry, prominent occiput, and tremors in the lower limbs. After several tests and evaluations, the final diagnosis was confirmed at three months old. Her family history indicates the possibility of hereditary hyperekplexia.

**Comments:**

The cases were compared with information obtained through a bibliographical review. Both patients presented several symptoms associated with hyperekplexia, including neurological symptoms such as increased startle response, convulsions, and hypertonia, which were alleviated with appropriate treatment. So far, combining multidisciplinary assistance with drug treatment, particularly anxiolytics and anticonvulsants, with clonazepam being the most used, has significantly contributed to both patients’ improved quality of life. However, physical symptoms, such as hip dislocation and clubfoot, require future surgical intervention.

## RESUMO

**Objetivo:**

Relatar dois casos de pacientes pediátricos com hiperecplexia do interior de São Paulo, Brasil.

**Descrição do caso:**

Duas pacientes, uma com três anos e seis meses e outra com cinco meses, são atendidas por uma unidade da APAE (Associação de Pais e Amigos dos Excepcionais) e foram diagnosticadas com hiperecplexia 1, um transtorno neurológico caracterizado por uma desordem de sobressalto excessivo. Casos de hiperecplexia podem ser divididos em três subgrupos: hereditário, esporádico e sintomático. Diversos especialistas examinaram a paciente 1 desde que tinha três semanas de vida, levando a duas hipóteses diagnósticas iniciais (encefalopatia crônica não progressiva infantil e paralisia cerebral espástica). Ela foi diagnosticada com hiperecplexia 1 aos onze meses, quando constatada uma mutação no gene GLRA1. A paciente 2, após o nascimento, apresentou hiperextensão de ambas as pernas, orelhas de implantação baixa, assimetria craniana, occipital proeminente e tremores nos membros inferiores. Aos três meses, após testes e avaliações, chegou-se ao diagnóstico final. O histórico familiar da paciente 2 indica a possibilidade de hiperecplexia hereditária.

**Comentários:**

Os casos foram comparados com informações obtidas por revisão bibliográfica. Ambas pacientes apresentaram vários sintomas associados à hiperecplexia, incluindo sintomas neurológicos, como resposta de sobressalto aumentada, convulsões e hipertonia, que foram amenizados com tratamento adequado. Até o momento, a combinação de assistência multidisciplinar com tratamento medicamentoso feito com ansiolíticos e anticonvulsivantes, sendo o clonazepam o medicamento mais utilizado, contribui significativamente para uma melhor qualidade de vida das pacientes. No entanto, sintomas físicos, como luxação de quadril e pé torto, requerem intervenção cirúrgica futura.

## INTRODUCTION

Hyperekplexia (HK) is an infrequent neurological disorder characterized by excessive startle responses noticed shortly after birth.^1,2^ It is a form of startle syndrome and can be divided into three main subgroups: hereditary, sporadic, and symptomatic.^3^ The genetic mutation in patients with this disease is often found in the α1 subunit of the glycine receptor, GLRA1.^4^ In addition to the GLRA1 gene, SLC6A5, GLRB, GPHN, and ARHGEF9 gene mutations have also been described.^5,6^ More than 200 confirmed cases have been reported worldwide.^7^

HK symptoms can be expressed in two forms: major and minor.^4,6^ The major form is characterized by an unusual startle reaction to sudden and unexpected noises or movements. If the patient is startled, they may experience head arching, jerky movements, or a rigid fall to the floor (without losing consciousness). The frequency and severity of the startle may be exacerbated by emotional tension, stress, or fatigue. The minor form typically manifests with exaggerated and inconsistent startle reactions but few or no other symptoms. In newborns, sudden death may occur due to laryngospasms and cardiorespiratory failure.^8^ In infants, the reaction may be triggered by fever. In both children and adults, the intensity of the startle response can be affected by stress or anxiety.

Symptoms vary widely, ranging from exaggerated startle reactions to episodes of apnea in babies and even accidents due to falls.^4,9^ Additionally, the disease may be associated with abdominal hernia, hip dislocation, developmental delays, and congenital clubfoot. HK diagnosis is based on clinical findings, molecular genetics that can identify mutation in genes, and electrophysiology tests. Electrophysiology tests include electromyography, which records electrical impulses produced by muscles, and electroencephalography, which records the electrical activity of the brain. Family history is also important due to genetic factors.

The disease’s differential diagnoses include symptomatic HK and spasticity, epilepsy associated with perinatal brain injury, and metabolic brain diseases. These can be excluded by a normal electroencephalogram (EEG) and when there is a reduction or abolition of rigidity and spasms during sleep. Currently, the treatment of choice for HK involves the use of anxiolytics and antispastic drugs, with clonazepam being the most prescribed medication, significantly reducing symptoms in most people.^9,10^ In children, low doses of medication are necessary, combined with physical exercise blocks rather than traditional physiotherapy or rigorous training.

Considering the low frequency of the disease, detailed studies on HK are necessary, including its signs, symptoms, treatment, and the importance of gearing health professionals and families toward early diagnosis and treatment to improve quality of life.

This study reports two cases of pediatric female patients with HK.

## CASE REPORT

Case 1. Patient 1 is a female, aged three years and six months, from a small city in São Paulo state, with around 36,000 inhabitants, located approximately 600 km from the state capital. At three weeks of age, she went to a health unit for a checkup, where the doctor suspected an alteration in her limbs but offered no syndromic diagnosis. She was referred to the Association of Parents and Friends of People with Disabilities (APAE, *Associação de Pais e Amigos dos Excepcionais*) for evaluation by a specialist to help define the diagnosis. At an APAE unit in the same city, she was examined by a physiotherapist, a speech therapist, a psychologist, a nurse, an occupational therapist, and a pediatrician. They found contractures in her hands, feet, and knees and alterations in her upper and lower limbs.

When she was around four months old, with a history of significant morphological changes, the pediatrician at APAE, suspecting childhood chronic non-progressive encephalopathy (the first diagnostic hypothesis), referred her to a geneticist, a pediatric cardiologist, a dermatologist, an orthopedist, an ophthalmologist, and a neurologist. The APAE pediatrician also referred her to the Association for Assistance to Children with Disabilities (AACD, *Associação de Assistência à Criança Deficiente*) due to severe spasticity.

**Figure 1:**
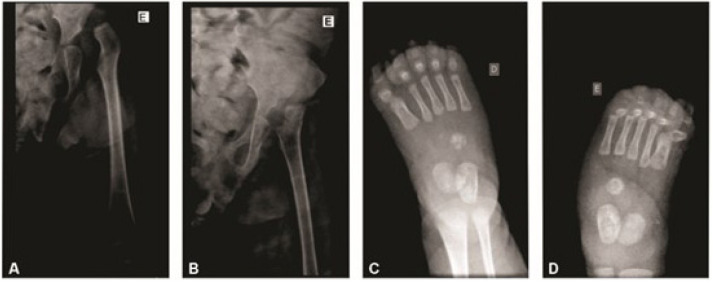
Radiographs of patient 1. (A-B) Left hip showing femoral head dislocation; and right (C) and left (D) feet showing structural changes in the hindfoot, midfoot, and forefoot regions.

The specialists found several orthopedic changes, including X-ray evidence of hip abnormalities (Figures 1 A and B): non-identification of the physis of the left femoral head and dislocation of the femoral head relative to the acetabular roof with a shallow appearance. Radiographs of both feet (Figures 1 C and D) revealed structural changes in the hindfoot, midfoot, and forefoot regions, indicative of congenital clubfoot.

The neurologist suspected spastic cerebral palsy (the second diagnostic hypothesis). One of the speech therapist’s findings was dysphagia associated with broncho-aspiration, in addition to significant failure to gain weight, with a percentile below 3. The mother reported two episodes of seizures, similar to absence seizures, with mild spasms in the upper and lower limbs.

Considering this clinical picture, a place was requested in the Project Loving Care for Children (*Projeto Amor de Criança*), a nonprofit organization specialized in helping children with cerebral palsy. The organization’s activities take place at an outpatient clinic of a university in a larger city, with a population of about 240,000 people, also far from the state capital but 142 km from the patient’s home city. It was there that doctors first investigated the possibility of a genetic syndrome.

Therefore, a genetic test was conducted, and at eleven months old, the patient was diagnosed with hyperekplexia 1, with changes in the GLRA1 gene, position chr5:151,859,888, C>T variation, where aspartate was replaced in codon 125 by asparagine, causing a genetic alteration. Following the test result, an EEG was performed, showing generalized epilepsy. The neurologist prescribed 2 ml of valproic acid (250 mg suspension) twice a day.

Currently, the patient shows improvement in her neuropsychomotor development and has not had seizures after using the prescribed medication. She is awaiting surgery to correct both feet.

Case 2. Patient 2 is also a female, currently five months old, from the same small city as patient 1. Her mother underwent prenatal care, during which she had a history of gestational hypertension, used methyldopa, and was affected by Bell’s palsy in the third trimester of pregnancy. Patient 2 was born by cesarean section with gestational age of 39 weeks and Apgar score of 4/8 at birth, presenting with a double nuchal cord and meconium aspiration. Immediately after birth, upon physical examination, she showed hyperextension of both legs, low-set ears, cranial asymmetry, prominent occiput, and tremors in the lower limbs during manipulation. For this reason, she was transferred to a larger city, the same mentioned in case 1, 142 km from the patient’s home city. There, she stayed in the neonatal intensive care unit (ICU) for 14 days.

An investigation was initiated with a head computed tomography scan, which found asymmetry of the lateral ventricles, with larger dimensions on the left side, slight dilation of the atrium and occipital horn, and persistence of the cavus septum pellucidum. After evaluation, a pediatric neurologist indicated treatment with phenobarbital 3 mg/kg/d due to the patient’s seizures presenting since birth. Additional tests were requested for further investigation, such as radiography for persistent stiffness after initial suspicion of arthrogryposis, head tomography without contrast, EEG, and karyotype. The EEG revealed abnormal results, with signs of diffuse irritative activity. The karyotype found hyperekplexia 1, with alteration in the GLRA1 gene, position chr5:151. 859,794, T>C variation, where tyrosine at position 156 was replaced by cysteine, which is especially harmful.

When investigating the family history, the patient’s mother reported that the paternal grandmother was also born with stiffness in her lower limbs. According to the grandmother, the patient’s father presented the same signs and symptoms as the newborn at birth; he was monitored at APAE from 14 days of age until he was 12 years old, had changes in the corpus callosum, and to this day, he has tremors in his lower limbs, though no syndrome was confirmed. The grandmother also mentioned that, according to family reports, her own mother, the patient’s paternal great-grandmother, had several miscarriages early in pregnancy. The patient’s maternal family presented no history of miscarriages or syndromes.

Currently, the patient continues to be assisted by APAE in her home city by a multidisciplinary team, which noticed a delay in neuropsychomotor development. The phenobarbital was replaced by clonazepam drops, a standard medication for relieving the HK symptoms. She is awaiting the results of complementary exams.

## DISCUSSION

Hyperekplexia causes complex genetic neurodevelopmental disorders characterized by an exaggerated startle reaction in infants and children, which may be linked to developmental delays or intellectual disability. These conditions are often caused by a congenital metabolic error or a brain malformation (with or without microcephaly or epilepsy) and are different from hereditary HK. An example of this is early infantile epileptic encephalopathy, characterized by epilepsy (often intractable focal seizures or febrile seizures and dysmorphic features). In the reported cases, we had neuropsychomotor delays associated with seizures, leading to a first diagnostic hypothesis of infantile encephalopathy.^11^ The literature indicates that clinical features help differentiate HK from epilepsy, such as short episodes (lasting only a few seconds), spared consciousness, the absence of other abnormal movements, and unexpected stimulus-inducing falling accidents.^5,9^

Studies show that most people with HK suffer from neonatal hypertonia and an exaggerated startle response, as was the case with the patients in our study.^9^ The literature also reveals that external abnormalities, such as hip dislocation, presented in patient 1, are common.^8,9^

There is one HK form that is hereditary. It is diagnosed by a three-generation family history, paying attention to family members with HK and considering relevant findings documented by direct examination or review of medical records, including the results of molecular genetic tests.^11^ As such, in the first reported case (patient 1), it was impossible to determine whether the variant was genetically inherited since the mother did not present the mutation, and it was impossible to look for the variant in the patient’s father and past generations. In the second case (patient 2), the karyotype of past generations is being carried out to confirm the diagnosis.

The reported patients use clonazepam, which, according to the literature,^9,10^ is considered a first-line therapy for HK because it enhances GABA-gated chloride channel function. Literature assumes it compensates for defective glycine-gated chloride channel function.^9^ Antiepileptic medications such as carbamazepine, phenytoin, valproic acid, and vigabatrin were also used to treat both patients, similarly recommended by the literature.^12^

After studying both patients’ medical histories and comparing them with the literature review, we concluded that with the assistance provided by APAE and the use of medication, the patients’ clinical condition and quality of life have improved. Patient 1 has significant skeletal changes, but the progress in motor development is notable. Regarding speech, she has a reduced lexical repertoire for her age. In patient 2, seizures have significantly improved.

Nevertheless, we believe that, due to the association of spasticity symptoms, motor limitations, the low frequency of the disease, and the few cases reported worldwide, the medical teams could not diagnose patient 1 earlier. The literature points out that HK can be misdiagnosed as epilepsy or, in older children, as disorders like tic, anxiety, or somatoform.^13^ In the first case, the doctors initially suspected childhood chronic non-progressive encephalopathy, then spastic cerebral palsy. This late diagnosis may have delayed early treatment. As for patient 2, a prompt diagnosis was likely possible due to hyperextension of the limbs at birth and early APAE monitoring and karyotyping.

Since the literature reiterates early diagnosis as fundamental, this report aims to raise pediatricians’ awareness about hyperekplexia and supports the notion that timely recognition can significantly mitigate the potential consequences of the disorder, enhancing patients’ overall quality of life. Therefore, we recommend, along with several authors, the nose-tap test, a helpful bedside test where an individual uses the finger to tap the nose of the patient in search of an exaggerated head retraction reflex.^14,15^

## Data Availability

The database that originated the article is available with the corresponding author.
